# Evaluation of a shared decision-making communication skills training for physicians treating patients with asthma: a mixed methods study using simulated patients

**DOI:** 10.1186/s12913-019-4445-y

**Published:** 2019-08-30

**Authors:** Evamaria Müller, Alice Diesing, Anke Rosahl, Isabelle Scholl, Martin Härter, Angela Buchholz

**Affiliations:** Department of Medical Psychology, University Medical Center Hamburg-Eppendorf, Martinistr. 52 (W26), D-20246 Hamburg, Germany

**Keywords:** Patient-centered care, Physician-patient relations, Education, Patient participation, Adherence

## Abstract

**Background:**

Shared decision-making (SDM) is a key principle in asthma management, but continues to be poorly implemented in routine care. This study aimed to evaluate the impact of a SDM communication skills training for physicians treating patients with asthma on the SDM behaviors of physicians, and to analyze physician views on the training.

**Methods:**

A mixed methods study with a partially mixed sequential equal status design was conducted to evaluate a 12 h SDM communication skills training for physicians treating patients with asthma. It included a short introductory talk, videotaped consultations with simulated asthma patients, video analysis in small group sessions, individual feedback, short presentations, group discussions, and practical exercises. The quantitative evaluation phase consisted of a before (t0) after (t1) comparison of SDM performance using the observer-rated OPTION^5^, the physician questionnaire SDM-Q-Doc, and the patient questionnaire SDM-Q-9, using dependent t-tests. The qualitative evaluation phase (t2) consisted of a content analysis of audiotaped and transcribed interviews.

**Results:**

Initially, 29 physicians participated in the study, 27 physicians provided quantitative data, and 22 physicians provided qualitative data for analysis. Quantitative results showed significantly improved performance in SDM following the training (t1) when compared with performance in SDM before the training (t0) (OPTION^5^: t (26) = − 5.16; *p* < 0.001) (SDM-Q-Doc: t (26) = − 4.39; *p* < 0.001) (SDM-Q-9: t (26) = − 5.86; *p* < 0.001). The qualitative evaluation showed that most physicians experienced a change in attitude and behavior after the training, and positively appraised the training program. Physicians considered simulated patient consultations, including feedback and video analysis, beneficial and suggested the future use of real patient consultations.

**Conclusion:**

The SDM communication skills training for physicians treating patients with asthma has potential to improve SDM performance, but would benefit from using real patient consultations.

## Background

An essential aspect of patient-centered care is shared decision-making (SDM). SDM describes the process in which clinicians and patients make a joint treatment decision based on the best current medical evidence and patient preferences. The process of SDM requires that both the patient and physician are aware of the decision-making situation, mutually share information, and identify themselves as equal partners in negotiating clinical decisions [1].

Despite strong international advocacy for SDM and increasing implementation efforts, SDM is not yet the norm in routine care [2, 3]. Although SDM can be applied in most decision-making situations, [4] it is especially relevant in the treatment of chronic and potentially life-threatening diseases, where multiple evidence-based treatment options exist, and treatment decisions might have long-term consequences for the patient [5, 6].

Bronchial asthma is a prevalent chronic respiratory disease. Current clinical guidelines for the management of patients with asthma recommend SDM as key principles in asthma management. [7] SDM also benefits the quality of life for pediatric patients with asthma, improves asthma control, [8] and delays exacerbations of asthma attacks [9]. Moreover, SDM improves pharmacotherapy adherence and clinical outcomes of adult patients with poorly controlled asthma, [10] and patient commitment to therapy [11]. Despite promising evidence and integration in clinical guidelines, patient-centered communication and SDM continue to be poorly implemented in the care of asthma patients [12, 13].

Implementation research intents to understand what kind of health care interventions work in “real world” settings, how they work, and why. Also, implementation research investigates approaches to improve these interventions [14]. Implementation research on SDM has recently shown that interventions on multiple system levels, including different stakeholders, are needed for a change of care that fully embraces SDM [3]. However, strategies to foster the implementation of SDM usually focus on clinician-mediated or patient-mediated interventions [15, 16]. SDM training also addresses the attitudes and consultation behaviors of clinicians, which are important factors in the advancement and uptake of SDM [17]. Still, many existing SDM training programs have not been evaluated, and there are few studies that have assessed the outcome of SDM training on clinician SDM behaviors [18, 19].

Currently, there is poor quality evidence on the efficacy of interventions that aim to improve clinician SDM behaviors, mainly because of the heterogeneity of SDM training programs, [18, 19] and a lack of consensus on how to assess the adoption of SDM [15, 16]. Studies that have investigated the concordance between different perspectives on SDM have provided inconsistent results. [20–25] Therefore, dyadic or triadic SDM measurement approaches including the perspectives of the clinician, the patient, and an independent observer are gaining increasing attention [26–28].

Quantitative analysis of the effectiveness of interventions to improve the SDM behaviors of physicians continues to produce inconclusive evidence [15]. This can be partly explained by a lack of consensus on training content, [29] and on evaluation outcomes. [17] However, explorative qualitative studies on the perspectives of clinicians might provide helpful information [30] to design or adapt effective SDM communication skills training. Mixed methods study designs combine the historically distinct approaches of quantitative and qualitative research and are relevant to provide data to address complex health care interventions, [31] including SDM communication skills training. Recently, a mixed methods study assessed the perceptions of clinicians and the barriers towards the adoption of SDM after training. The results provided valuable input for the design of future training and implementation strategies [32]. Implementation research has highlighted the importance of eliciting the perspectives of stakeholders [33] to foster the implementation of SDM, [34] and clinicians are key stakeholders in this process.

## Methods

### Aim, design and setting of the study

This study aimed to evaluate the impact of SDM communication skills training for physicians treating patients with asthma on the SDM behaviors of physicians measured from three different perspectives (observer, physician, patient). Additionally, this study aimed to analyze physician views on the training, including their perceptions of intrapersonal change after the training, and their suggestions for improvement of the training. To meet these aims, a mixed methods study with a partially mixed sequential equal status design was conducted.

**Fig. 1:**
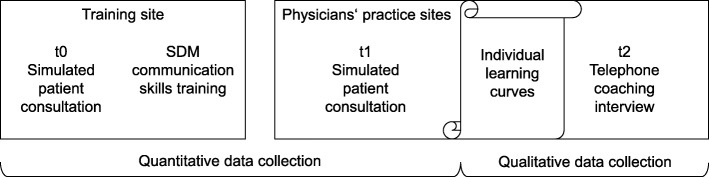
Study design. SDM = shared decision-making

The present study was undertaken with the cooperation of Mundipharma GmbH [32]. It was conducted following a partially mixed sequential equal status design, [35] in line with the guidelines for conducting and reporting mixed analysis studies [36]. The study design included a quantitative evaluation phase using a pre-post design with no control group and a qualitative evaluation phase, as shown in Fig. 1. Study subjects were physicians treating asthma patients in outpatient care in Germany. Cooperating partners of Mundipharma GmbH invited physicians nationwide, organized their training, and were present at the training sites in Berlin, Hamburg, and Munich in Germany. Authors who were members of the study team conducted the physician training (EM, IS, MH and AB), and the coaching interviews (EM). The training sessions were independent of study participation. Non-probability sequential sampling was used with a parallel study design that included a convenience sample of physicians who were willing to participate in the study. All participating physicians signed an informed consent form including permission for pseudonymized data analysis. The study was conducted in accordance with the Code of Ethics of the Declaration of Helsinki and was approved by the Ethics Committee of the Medical Association of Hamburg, Germany (Registration No: PV4973).

### SDM training and evaluation

The SDM communication skills training consisted of 12 h of training administered during a period of 2 days on one weekend. At the beginning of the training, participating physicians performed videotaped consultations with simulated asthma patients (psychology and health sciences students), which were subsequently analyzed in small group sessions during the training. Moreover, the training included a short introductory talk by a pulmonologist, individual feedback by the simulated patients, fellow participants and trainers, short presentations, group discussions, and practical exercises with fellow participants. The training focused on SDM skills based on the three-talk model for clinical practice, [37] but also included patient-centered communication and motivational interviewing skills [38]. After the training, participating physicians had the opportunity to perform a second videotaped consultation with a simulated asthma patient at the physician’s practice site.

Participating physicians received written individual learning curve results comparing their consultations before and after the training. Learning curves included feedback on how much time simulated patients had to state their concerns and physician’s proportions of speech. The feedback also included a comparison of the physician’s and simulated patient’s perceptions of SDM during the consultations. Additionally, the physicians received individual feedback and advice based on the observer-rated Four Habits Coding Scheme, [39] a template for both guiding and measuring the communication behaviors of physicians [40].

In subsequent individual semi-structured telephone coaching interviews, one team member (EM) explained individual learning curves to each participant and explored perceptions of the training using an interview guideline. First, the understanding and perception of the learning curves were discussed and time was provided to voice concerns, criticism, and needs regarding the content of the training or the physician’s clinical practice. In the second part of the telephone call, physicians were asked: whether they noticed changes in attitudes and communication behaviors after the training; how satisfied they were with the training program and its components; which training components benefited them the most; how they rated the training program; if they would recommend the training to a friend or colleague; and if they had suggestions on how to improve the training program.

### Data collection, measurement, and outcomes

#### Quantitative evaluation phase

Data were collected from May 2015 until December 2016. Demographic and professional characteristics of the participating physicians were collected at the beginning of the training program. Further quantitative data were collected at the beginning of the training (t0) and after the training at the physician’s practice site (t1). Data collection was equivalent at both measurement points. Due to the design of the study, blinding was not feasible.

Before the training, four asthma patient case vignettes with identical airflow parameters. They were developed by study team members (EM, AB) experienced in the design of medical case vignettes, and revised by an experienced pulmonologist. Two vignettes were used in simulated patient consultations at t0 and another two were used at t1. Simulated patients were psychology and health sciences students who had received 2 h of training. Consultations with simulated patients were videotaped and transcribed verbatim. Two trained team members independently evaluated the videotapes and transcripts with the OPTION^5^ scale (primary outcome) [41]. Training in the application of the OPTION^5^ consisted of independent rating of ten audiotapes and transcribed patient and physician consultations and subsequent comparison and discussion of the results, until consensus was reached. The OPTION^5^ scale is a psychometrically tested five-item observer-based instrument measuring the physician’s efforts to involve the patient in the SDM process using a five-point Likert scale (0 = not observed; 4 = executed to a high standard). Simulated patients were included to measure SDM from the patient’s point of view. Directly after the consultations, the simulated patients completed the patient version of the nine-item Shared Decision Making Questionnaire (SDM-Q-9) [42] and physicians completed the physician version of the nine-item Shared Decision Making Questionnaire (SDM-Q-Doc) [43] (secondary outcomes). The SDM-Q-Doc and the SDM-Q-9 are both psychometrically tested nine-item self-reporting instruments measuring the physician and patient perspective on the decision-making process using a six-point Likert scale (0 = completely disagree; 5 = completely agree).

#### Qualitative evaluation phase

Data were collected from October 2015 until February 2017. Physicians were phoned or e-mailed to make appointments for the telephone coaching interviews (t2). They were sent learning curves by mail prior to the interviews. One team member (EM) conducted and audiotaped semi-structured telephone coaching interviews using an interview guideline, as described above.

### Data analysis

#### Statistical analysis of the quantitative evaluation phase

Descriptive statistics were used to characterize the study sample and the simulated patient consultations (t0 and t1). To establish the inter-rater reliability of the OPTION^5^ scores, an intra-class correlation coefficient (ICC) using a one-way random effects model was calculated. The ICC was calculated to test if the mean of the two ratings was suitable for further analysis [44]. The means of individual item scores were used to replace missing values for the SDM-Q-Doc and SDM-Q-9 for up to two random missing values per case [42]. Histograms were visually inspected, and p-p plots were used to test whether the data concurred with the assumption of normality of difference of the dependent t-test. To test whether the physicians showed more SDM behaviors following the training, the mean differences were analyzed between the pre-and post-measurements of OPTION^5^, the SDM-Q-Doc, and the SDM-Q-9 using dependent t-tests. Since previous training studies showed an increase in SDM behaviors after training, it was assumed that higher scores occurred at the second measurement point (t1), and one-tailed t-tests with an alpha level of 0.05 were used. Due to the pragmatic setting of the study, we did not perform a power-analysis prior to the study, but calculated a post-hoc power analysis with g*power.

#### Analysis of the qualitative evaluation phase

Descriptive statistics were analyzed to characterize the telephone coaching interviews (t2). Audiotapes of interviews were transcribed verbatim, and the transcripts were pseudonymized and imported to MAXQDA software version 10 (VERBI, GmbH, Berlin, Germany), which is a software supporting qualitative and mixed methods analysis. Transcripts were analyzed following the principles of directed content analysis, as described by Hsieh and Shannon [45]. One team member (AR) made herself familiar with the data, deduced the main codes from the interview guideline, and developed subcodes from the data inductively. As coding entities, units of meaning were chosen. Two team members (AR and AD) independently coded seven (30%) randomly selected transcripts to test the suitability of the code system. The codes were discussed with a third team member (EM) and adapted if necessary. After establishing sufficient consistency, one team member (AR) coded the entire data set and discussed any uncertainties with another team member (EM).

## Results

### Sample characteristics

**Fig. 2:**
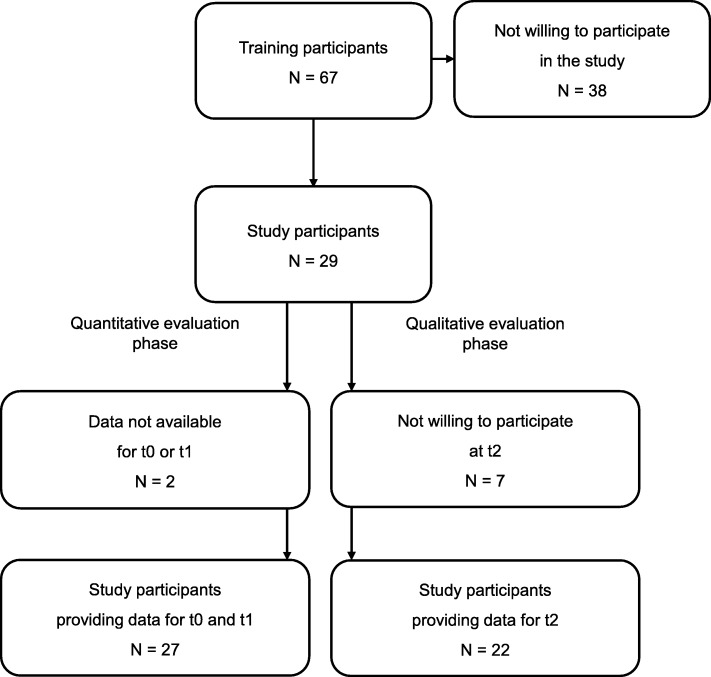
Flow diagram of training and study participants

Seven training sessions were conducted in 2015–2016, which took place in Hamburg, Berlin, and Munich, Germany. Out of 67 physicians who participated in the training sessions, 29 physicians agreed to participate in the study. Reasons for non-participation were unknown. Table 1 shows the study sample characteristics. Figure 2 displays the flow diagram of the training and study participants. As two study participants provided data for t0 or t1 only, 27 physicians were included in the quantitative data analysis. The mean and standard deviation (SD) of the duration of the simulated patient consultations at t0 was 8.64 ± 2.27 min (range: 4.9–14.3 min). Simulated patient consultations at t1 occurred at a mean of 62 ± 33.14 days (range: 19–160 days) after the training, and had a mean duration of 11.09 ± 3.51 min (range: 5.55–21.78 min). Out of 29 study participants, 22 participated in the telephone coaching interviews (t2). Seven physicians did not participate due to time constraints or for unknown reasons. Telephone coaching interviews took place 149 ± 64.67 days (range: 56–272 days) after the second simulated patient consultation (t1) and with a mean duration of 35.95 ± 17.19 min (range: 8.43–86.33 min).

**Table 1:** Characteristics of the study sample

	Study participants (*N* = 29)
Gender	
Male (N) (%)	21 (72%)
Female (N) (%)	8 (28%)
Age in years	
Mean (± SD)	56.48 (± 4.47)
Range	49–67
Type of practice	
Single practice (N) (%)	11 (38%)
Joint practice (N) (%)	18 (62%)
Work time model	
Full-time (N) (%)	25 (86%)
Part-time (n) (%)	4 (14%)
Medical background	
Pulmonology (N) (%)	14 (48%)
Primary care (N) (%)	6 (21%)
Pediatrics (N) (%)	2 (7%)
Missing (N) (%)	7 (24%)
Years of professional experience	
Mean (± SD)	28.18 (± 6.47)

*Abbreviations*: *SD* standard deviations

### Results from the quantitative evaluation phase

**Fig. 3:**
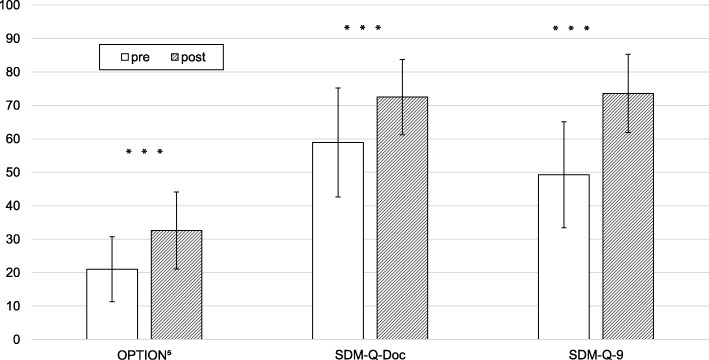
Shared decision-making behaviors before and after the training. Error bars represent the standard deviations (SD) from the mean. *** = *p* < .001. OPTION^5^ = observer-rated SDM. SDM-Q-Doc = physician SDM questionnaire. SDM-Q-9 = patient SDM questionnaire

There was a moderate degree of reliability with an average ICC of 0.52 and a 95% confidence interval (CI) from − 0.02 to 0.77 for the OPTION^5^ scores. The mean of two raters’ OPTION^5^ scores underwent further analysis. As visual evaluation of the histograms and p-p probability plots of the data supported the assumption of normality of difference of the scores, dependent t-tests were calculated to compare mean scores of the OPTION^5^ (primary outcome), the SDM-Q-Doc, and the SDM-Q-9 (secondary outcomes) data before (t0) and after (t1) the training (Fig. 3). From the observer’s point of view, physicians displayed more SDM behaviors at the second measurement point t1 (t (26) = − 5.16; *p* < .001). The mean of the OPTION^5^ increased by 11.57 ± 11.65 points on a scale from 0 to 100 from a mean of 21.02 ± 9.71 points before the training to a mean of 32.59 ± 11.49 points after the training. Physicians rated their own SDM behaviors as higher at the second measurement point t1 (t (26) = − 4.39; *p* < .001). The mean score of the SDM-Q-Doc increased by 13.58 ± 16.06 points on a scale from 0 to 100 from a mean of 58.93 ± 16.26 points to a mean of 72.51 ± 11.27 points. Simulated patients rated the physician SDM behaviors higher at the second measurement point t1 (t (26) = − 5.86; *p* < .001). The mean score of the SDM-Q-9 increased by 24.28 ± 21.54 points on a scale from 0 to 100 from a mean of 49.30 ± 15.86 points to a mean of 73.58 ± 11.70 points. Post-hoc power analysis for the use of one-tailored dependent t-tests (*n* = 27, α = .05) resulted in a power of .90 for large effects (d = .50).

### Results from the qualitative evaluation phase

#### Experience of change in attitudes and communication behaviors

Some physicians experienced hardly any change due to the SDM training, but most participating physicians found that they consciously paid more attention to their communication behaviors and were more self-reflective since the SDM communication skills training (Table 2). Some physicians detected a more patient-centered approach in their consultations since the training and found themselves paying attention to the patient’s perspective more often than before. Aiming for better understanding of patient concerns, some physicians asked more open questions, interrupted less, and asked patients for additional questions.

**Table 2:** Results from the qualitative evaluation phase

Main code	Exemplary quote
*Experience of change in attitudes and communication behaviors*	“I pay more attention to the way I steer my consultations. Previously, I have probably paid no attention to this, at all, in stressful situations. But now I try to reflect: Was this ok, the way I steered this consultation?” (P4)
*Satisfaction with the training and its components*	„I am grateful. It was a great opportunity to perform these [simulated patient] situations and receive supervision. You hardly ever get this opportunity as a physician in outpatient care or in hospital.“(P8)
*The most beneficial training components*	„I would say, analysis [of simulated patient consultations] in general and then the video-analysis, during which you see your own reactions and how you behave, and receive feedback from others watching this.” (P19)
*Recommendation to a friend or colleague*	„It depends on the person and his or her level of training. I would recommend it to beginners, but not necessarily to the more experienced.” (P20)
*Suggestions for improvement of the training*	„You know, to get the picture, you would have to accompany a physician during his consultation hours. You see, a simulated consultation with an actor is beneficial in some way, but it doesn’t correspond to the reality we have to face.” (P4)

P1-P22 in brackets = physicians who participated in the qualitative evaluation

#### Satisfaction with the training and its components

Overall, physicians appreciated the training and felt they benefited from participation. However, not all physicians agreed with all the SDM training contents and questioned the feasibility of some concepts. Some physicians felt that current time pressure in outpatient care prevents the communication ideals of asking open- questions. One physician recounted negative reactions to his attempts to involve patients in decision-making. Despite these critical opinions regarding the applicability of SDM in practice, most physicians expressed their approval of the training.

In general, physicians liked the short presentations and found them interesting, although a few mentioned they already knew some of the content. Above all, physicians praised the opportunity to receive supervision and multi-perspective feedback on their communication behaviors (Table 2). Physicians appreciated the positive and respectful feedback climate and the diverse feedback from simulated patients, colleagues, and trainers, all of them providing different points of view. Repeatedly, physicians valued the chance to learn from their peers’ simulated patient consultations. Mainly, physicians viewed the simulated patient consultations as beneficial and educational, although some of them did not feel comfortable performing an artificial consultation in front of an audience while being videotaped. However, most physicians valued video-analysis of simulated patient consultations as exciting, interesting, and helpful for self-reflection. In contrast to the overall appreciative judgment of video-analysis, a few physicians felt uncomfortable or stressed during the video-analysis, which made them think that they were undergoing a test themselves.

#### The most beneficial training components

In summary, most physicians considered simulated patient consultations and their subsequent reflection and video-analysis the most beneficial training components. However, physicians highlighted different aspects. Some physicians especially valued the chance to see their own reactions and non-verbal communication during video-analysis or gained most from receiving diverse feedback on their communication behaviors (Table 2). However, a few physicians could not pinpoint a central training component, but they experienced the succession of components as important. Other physicians gained most from the telephone coaching interviews including detailed analysis of individual learning curves.

#### Recommendation to a friend or colleague

All physicians asked if they would recommend the training program to a friend or colleague agreed. However, some physicians limited their consent to people they considered interested in the topic or to younger physicians just starting clinical practice (Table 2).

#### Suggestions for improvement of the training

To improve the training, some physicians felt that assessing communication behaviors under real-world conditions would be better (Table 2). Physicians suggested performing the coaching as an audit and feedback session at the practice sites and repeatedly expressed the need to integrate real patients into the training. Other physicians wanted the simulated patient consultations to be more realistic and reflecting their hectic multi-tasking practice environments. In line with this, physicians suggested integration of more diverse and more demanding patient examples. Some physicians felt the need introduce more practical exercises in the training and to have an exemplary video that illustrates optimal physician communication behaviors. Physicians also provided organizational recommendations, for examples, to reduce time intervals between the training components, to extend the training dates, to limit small group sessions to the second day of the training, and to provide references for self-study.

## Discussion

A mixed methods study was undertaken to analyze the impact of shared decision-making (SDM) communication skills training for physicians treating patients with asthma on the SDM behaviors of physicians, and to gain information about its reception and needs for modification. Study participants were predominantly highly experienced male physicians working full-time in outpatient care. The results from the quantitative evaluation phase showed increased results from all three perspectives, the physician, the patient, and the observer, after the training. The average SDM observer-rated OPTION^5^ score, the primary outcome, remained relatively low when compared with the relatively high physician and simulated patient SDM ratings. The results from the qualitative evaluation phase revealed that most physicians reported a change of attitudes and behaviors, and mainly approved the training and its components. Physicians considered simulated patient consultations, including subsequent feedback and video-analysis, most beneficial. Moreover, they suggested the integration of real or more realistic simulated patient consultations or the inclusion of actual patients with chronic asthma.

The results from the quantitative evaluation phase showed that physicians improved their SDM behaviors after the training sessions, indicating the potential for SDM communication skills training to support the implementation of SDM in practice. Physicians and simulated patients judged the physician consultation behaviors as highly participatory. Lack of blinding to the study conditions and social desirability bias may explain these high ratings, but the average scores of the SDM-Q-9 and the SDM-Q-Doc in this study are comparable to those found in previously published studies, [25, 46] indicating a common and reproducible finding. Similar to previous studies, [21, 25] OPTION^5^ scores were relatively low compared to the physician and simulated patient SDM ratings, which indicated a lack of consistency in measuring SDM from different viewpoints. However, rather than interpreting this as a problem and aiming to reduce these inconsistencies, systematic integration of all three perspectives may provide the most accurate picture of the SDM process [23].

In the qualitative evaluation phase, physicians voiced their overall satisfaction with the training and stated they would recommend it, especially to interested and younger colleagues. Participating physicians appreciated the opportunity to receive diverse multi-perspective feedback to analyze their own communication behaviors during video-analysis. They also valued the opportunity to watch their colleagues perform consultations. Although a few physicians expressed some discomfort in the training process, most physicians considered these training components to be beneficial and educational. Physicians reported changes in attitudes and behaviors reflecting relational skills, which is one of two core SDM competency categories agreed on by an international group of experts [29]. There were few criticisms of the training, which may be due to the sample of voluntary participants, and the fact that one of the trainers conducted the coaching interviews. This fact is likely to have introduced a social desirability bias. However, a few physicians expressed reluctance to incorporate SDM in daily practice as they considered it incompatible with the limited time in their busy practice schedules, which is a common argument against the use of SDM [47, 48].

Our study has several notable limitations and strengths. First, an easily accessible sample of physicians were recruited who were willing to participate in the study. This meant that it was likely that the study sample largely consists of physicians with a particularly positive attitude towards SDM training, [49] and who might not have been representative for physicians treating patients with asthma in outpatient care in Germany [50]. Moreover, most participating physicians were male, and this may have introduced a gender bias in the results of this study. The study sample was relatively small despite nationwide recruitment through Mundipharma GmbH, and there was no information regarding the reasons for non-participation in the SDM training or the study, and because of this, physician selection bias was likely. Due to the convenience sampling strategy, we did not calculate a power-analysis prior to the study, but performed a post-hoc power-analysis. Second, there was a lack of study blinding of the physicians, simulated patients, and observers, which might have introduced measurement bias due to the Hawthorne effect and social desirability bias. Observer study blinding would have been possible if the study analysis had been limited to analysis of the transcript data, but it was considered to be important to use videotapes for the OPTION^5^ ratings. Third, coaching interviews were conducted by one of the trainers, which might have introduced bias. Moreover, no behavioral theory was used for the quantitative analysis of the interviews, which is another limitation of this study. Fourth, the use of videotaped consultations with student volunteers as patient surrogates for quantitative evaluation limited the external validity of the findings as transference to routine practice was not measured.

However, the use of simulated patients is also a strength of this study as it allowed investigation of physicians’ SDM performance of the physicians under equivalent circumstances, [20, 51] increasing the internal validity of the study. Another important strength of the study was the combination of a quantitative and qualitative evaluation in one study, which allowed comparison of the measured effects of the training with perceptions of the involved physicians, to obtain information regarding effective training components and any needs for modification or change. Another possible strength of the study was that change in the SDM behaviors of the participating physicians was evaluated from three perspectives, which followed the recommendation of a triadic approach to measuring SDM (observer, physician, patient) [26, 28, 52]. Finally, this study also included established measures with promising psychometric properties [41–43, 53].

## Conclusion

This study showed that SDM communication skills training for physicians treating patients with asthma has the potential to improve SDM performance, but would benefit from using real patient consultations. This study combined quantitative and qualitative methods, which allowed for a more comprehensive evaluation of the training compared to one methodological approach alone. Results from both evaluation phases indicate the potential to initiate change in the attitudes and behaviors of physicians. However, physician-reported changes in interviews do not imply implementation of SDM but reflect relational skills, a category of SDM core competencies considered essential by experts in the field [29]. It is conceivable that physicians need opportunities to reflect more on their daily practice and learn basic patient-centered communication skills before aiming to implement SDM [37]. Ongoing training including small group sessions, video analysis of real patient consultations, more opportunities for role-play, audit and feedback sessions at the practice sites could improve the transfer of newly acquired skills to daily clinical practice [54]. These ideas were also discussed by some physicians in this study. The introduction of patient-mediated interventions, including decision aids or initiatives such as the ‘Ask 3 Questions’ campaign [55] could complement the training. To achieve implementation in routine care, complex SDM interventions targeting both healthcare providers and patients have so far resulted in the most promising results [16].

## Data Availability

The data (German language) that support the findings of this study are available on request from the corresponding author [EM]. The data are not publicly available, because consent for publication of raw data was not obtained, and the dataset could pose a threat to confidentiality and compromise research participant privacy and consent. Research participants agreed to participate in the study under the condition that raw data were not made available to the public.

## Abbreviations

ICC: Intra-class correlation coefficient
SDM: Shared decision-making
